# Association between sociodemographic factors and health beliefs related to breast cancer screening behavior among Northern Thai women: a hospital-based study

**DOI:** 10.1038/s41598-024-58155-y

**Published:** 2024-03-31

**Authors:** Surin Jiraniramai, Kanokporn Pinyopornpanish, Nahathai Wongpakaran, Chaisiri Angkurawaranon, Victoria L. Champion, Imjai Chitapanarux, Wichuda Jiraporncharoen, Tinakon Wongpakaran

**Affiliations:** 1 Department of Family Medicine, Faculty of Medicine, Chiang Mai University, Chiang Mai, Thailand; 2 Global Health and Chronic Conditions Research Group, Chiang Mai University, Chiang Mai, 50200 Thailand; 3 Department of Psychiatry, Faculty of Medicine, Chiang Mai University, 110 Inthawarorot Rd., Sriphum, Muang, Chiang Mai, 50200 Thailand; 4 School of Nursing, Indiana University, Indianapolis, IN 46202 USA; 5 Melvin and Bren Simon Comprehensive Cancer Center, Indiana University, Indianapolis, IN 46202 USA; 6 Department of Radiology, Faculty of Medicine, Chiang Mai University, Chiang Mai, Thailand

**Keywords:** Breast cancer, Screening, Health beliefs, Perception, Health services, Public health

## Abstract

Early diagnosis of breast cancer is crucial for reducing mortality rates. The purpose of this study is to determine the impact of demographics/social determinants of health on beliefs about the practice of self-breast examination, using mammogram and ultrasound in the context of breast cancer screening among Thai women in a hospital-based setting for implying program planning and future research. A cross-sectional study was conducted in two health centers in Chiang Mai Province from August 2021 to December 2021, involving 130 Thai women ages 40 to 70 years. Data were collected by a survey using a questionnaire to gather sociodemographic information, and health beliefs about breast cancer and screening behavior utilizing the modified Thai version of Champion's Health Belief Model Scale (MT-CHBMS). Descriptive statistics, t-tests, ANOVA, and linear regression models were employed for examining association between sociodemographic factors and health beliefs about the practice of self-breast examination (BSE), using mammogram (MG) and ultrasound (UTS). Health insurance schemes were associated with Benefit-MG, Barrier-BSE, Barrier-MG and Barrier-UTS subscales. Additionally, monthly income was associated with Barrier-MG and Barrier-UTS subscales. The most common barriers reported were “embarrassment”, “worry”, and “takes too much time”. To enhance breast cancer screening in Thailand, program planning and future research should focus on health insurance schemes, especially women with social security schemes, as they may be the most appropriate target group for intervention.

## Introduction

Female breast cancer is the most commonly diagnosed cancer, with approximately 2.3 million new cases and 685,000 deaths reported in 2020^1^. It is the leading or second leading cause of female cancer-related deaths in 95% of countries worldwide^2^. In 2022, breast cancer in Thailand accounted for 38,559 cases^3^, making it the most prevalent female cancer, and accounting for 32.64% of the top five cancers in the northern region of Thailand^4^. This region has been predicted to have the highest age-standardized incidence rate (ASR) and proportion of female cancer cases by 2025^5^. However, early diagnosis and treatment can significantly reduce breast cancer mortality rates and improve women's overall health^6^.

Globally, high-income countries have adopted mammography as the standard screening method for early diagnosis of breast cancer, which helps reduce rates of advanced and fatal breast cancer^7^. In contrast, low to moderate-income countries, including Thailand^8–10^, often rely on breast self-examination (BSE) due to its insufficient mammography resources, although it is considered less reliable^11–13^. Therefore, it is recommended that women regularly and accurately perform BSE and consult with their physicians, who might recommend mammography and/or ultrasound if a lump is found^9^. It's important to note that BSE alone is not an effective method for reducing breast cancer mortality^14^. However, a recent population-based study of 1,906,697 women participating in a breast cancer awareness program in Thailand reported that women who regularly practiced BSE had better survival rates compared to non-practicing women. Additionally, a significantly higher proportion of smaller tumor sizes and earlier stages of breast cancer were observed in the group that regularly performed BSE. This positive outcome was attributed to the strong collaboration between village health volunteers and the use of BSE record booklets. Village health volunteers played a vital role in reminding women to perform BSE consistently, while the BSE record booklets helped women accurately follow the instructions and document their BSE practices^15^. Many countries of low to moderate-income countries have BSE practice as the first line screening because it is easy, convenient, private safe and no specific equipment requirement. Its purpose is to make women familiar with both the appearance and feel of their breasts as early as possible, so that they will be able to easily detect changes in their breast^13,16^. The more practice of BSE, the more empower women health^8,13,17^ Based on these evidence, initial BSE is deemed appropriate for Thailand as a low to moderate-income country. The practice of BSE among women is influenced by their knowledge and beliefs about breast cancer and screening methods^17^.

In Thailand, the current guidelines for breast cancer screening^18^ include breast cancer screening according to age. For ages 20–39 years old, it is recommended that breast self-examination should be performed once a month. Women between 40 and 69 years should be examined by a doctor annually. If abnormalities are identified, a mammogram will be scheduled. For the age of 70 years old and over, mammography for breast cancer screening should be weighed in terms of benefits and risks based on individual’s life expectancy and preference. However, in the voluntary case of populations who wish to have breast cancer screening by mammogram in the first place, recommendations for screening have been added that are similar to those recommended by the American Cancer Society. This recommendation was caused by public health policy and public finance management in Thailand.

In some resource-limited areas, breast ultrasound has been proposed as a possible alternative for mammography in breast cancer screening because it is portable, less expensive than mammography, and versatile across a wider range of clinical applications. The use of ultrasound as an effective primary detection tool for breast cancer may be beneficial in low-resource settings where mammography is unavailable^19^. Furthermore, according to the findings of a multi-center randomized trial comparing ultrasound vs. mammography for screening breast cancer in high-risk Chinese women, ultrasound was superior to mammography for screening breast cancer in this group^19^. In Thailand, mammography is not available in most rural areas. Similarly, Thai women, like Chinese women, have smaller and denser breasts than Western women^20^. Additionally, ultrasound yields less pain or discomfort than a mammogram, which is one of the main problems preventing women from breast cancer screening.^21^.

In real-world practice, BSE is not widely adopted among most Thai women. From secondary data of the 2007 Health and Welfare Survey that comprised 18,474 women aged 20 years and older and the 2009 Reproductive Health Survey that comprised 26,951 women aged 30 to 59 years show that only 18.4% of women practice monthly BSE^21^, indicating a low level of knowledge and awareness about breast cancer and the importance of BSE, mammography, and ultrasound screening that are the steps for increasing diagnosis of breast cancer. Before planning effective interventions to motivate the use of these screening methods, it is important to understand Thai women's knowledge and beliefs about breast cancer screening. Previous studies have shown that the Health Belief Model is a reliable and valid tool for measuring individuals' knowledge and beliefs about breast cancer and screening methods^22^. This model predicts the behaviors of people who take action to prevent, screen for, or control illness conditions based on their personal beliefs or perceptions about a disease^23^. Champion's Health Belief Model Scale (CHBMS) is the first and most widely used tool in the literature across continents, countries, cultures, and ethnicities to measure women's beliefs about breast cancer screening^8,24–29^.

The CHBMS comprises six main constructs: susceptibility, seriousness, benefits, barriers, health motivation, and confidence (self-efficacy). This scale has also been developed to assess perceived benefits and barriers of BSE and mammogram screening^25–27,29–31^. Recently, a modified Thai version of Champion's Health Belief Model Scale (MT-CHBMS)^32^ incorporated ultrasound items for breast cancer screening. The primary reason for this addition is that ultrasound can effectively detect small and dense tissue tumors, particularly in younger Asian women who tend to have denser breast tissue compared to Western women^19^. In terms of advanced technology, techniques such as artificial intelligence (e.g., deep-learning-enabled clinical decision support systems) and classification of ultrasound images have demonstrated superior accuracy in detecting breast cancers compared to various screening tools currently available^33,34^. The MT-CHBMS has been found to be valid and reliable among Thai women^32^. This scale can be comparing perceived benefits and barriers of BSE, mammogram and ultrasound screening from associate predictors of sociodemographic factors. These predictors could be implying the program design for increasing breast cancer screening.

Numerous studies have demonstrated the significant impact of sociodemographic factors on women's breast cancer screening behaviors, with results varying across cultures and values. For instance, research conducted in Middle Eastern countries revealed notable associations between age, title, giving birth, BC screening in the last 6 months, BSE training, chronic disease, mental illness, and BSE practice^35^. Conversely, a study in a similar cultural context showed that BSE and mammography practices among women were influenced by the only level of their knowledge about breast cancer^36^. In an African country, a study found significant associations between income status, marital status, age of first childbirth in the family, and perceived susceptibility, health motivation, convenience, perceived benefits, and self-efficacy for BSE^37^.

Despite these findings, there is currently a lack of information regarding the health perception of Thai women, the scope of their health beliefs, and how demographics/social determinants impact these beliefs. Additionally, these results have been integrated to plan for detecting and managing for breast cancer in primary care of hospital that is the one of strategic in Thailand’s sustainable development goals^38,39^. Therefore, the objective of this study is to determine the impact of demographics/social determinants of health on beliefs about the practice of self-breast examination, using mammogram and ultrasound in the context of breast cancer screening among Thai women in a hospital-based setting for implying program planning and future research.

## Methods

### Study design and participants

A cross-sectional study was conducted in Chiang Mai province, Kingdom of Thailand, from August 2021 to December 2021. One hundred and thirty participants recruited with convenience sampling method for the study, consisting of women from two health centers: Maharaj Nakorn Chiang Mai Hospital, located in an urban area, and San Pa Tong Hospital, situated in a rural area. A comprehensive description of the development of the MT-CHBMS has been previously published^32^.

### Inclusion and exclusion criteria

The inclusion criteria for the study were as follows: individuals between the ages of 40 and 70 years (the recommended age for mammograms), no prior history of breast cancer or any other types of cancer, and not currently pregnant or breastfeeding. The exclusion criteria included individuals who were unable to communicate effectively due to language barriers and those who expressed unwillingness to complete the questionnaires.

#### Sample size

Sample size is calculated based on the following criteria.

Anticipated effect size (*f*^2^) was 0.15 (small). The desired statistical power level was 0.8

The number of predictors was 5. Therefore, the minimum required sample was 91. We recruited 130 participants for this study, indicating that it was sufficient.

### The data collection tools

To collect data at the outpatient clinic, the researchers gathered socio-economic information by structured interviewing. The questions included items such as age, religion, marital status, education level, healthcare insurance schemes (including the three main public health insurance schemes: government or state enterprise officer, social security scheme, and universal coverage scheme), income, and residential area. Then paper questionnaires were provided to all participants. Prior to completing the questionnaires, all participants provided written informed consent.

The questionnaire addressing beliefs was the MT-CHBMS. The CHBMS was translated into Thai, validated by a panel of experts, back translated, modified by adding content about ultrasound for screening breast cancer, and pretested. Confirmatory factor analysis was used with a sample of 130 Thai women aged 40 to 70 years old. The scales were measured with an ordinal scale using a five-point Likert type 1: “Strongly disagree”, to 5: “Strongly agree”. Each subscale can be used independently. In the case of overall assessment of the awareness of breast cancer and screening methods, the total score can be adopted but the questions concerning barriers must be reversed before summing up.

The MT-CHBMS’s Cronbach’s alphas values were acceptable, ranging from 0.74 to 0.93 for the scales)and valid(Content validity using the CVI index from 3 experts showed that the average Item-CVI was 1.00, all factor loading coefficients in the confirmatory factor analysis were significant(p < 0.001) and ranged from 0.413 to 1.029) tool for measuring the Health Belief Model related to the practice of breast self-examination (BSE), as well as investigating attitudes towards mammograms and ultrasounds^32^. The confirmatory factor analysis results of the CHBMS and MT-CHBMS. Each item had sufficient factor loadings (estimated coefficients) on the designated factor. All factor loading coefficients were significant (*p* < 0.001) and ranged from 0.413 to 1.029. The fit statistics were assessed to demonstrate how well the CFA model fitted the data. For the model MT-CHBM: chi-square = 2488.868, df = 1879, chi-square/df = 1.324, TLI = 0.961, CFI = 0.964, and RMSEA (90% CI) = 0.050(0.045–0.055). Except for the motivation subscale, 21 pairs of error terms in each subscale of T-CHBMS and 23 pairs of error terms of MT-CHBMS were correlated. All these error terms suggested a high correlation between items and became the potential sources of the model misfit.

The questionnaire consisted of 64 items distributed among 10 subscales: susceptibility (5 items), seriousness (7 items), benefits of BSE (6 items), barriers to BSE (6 items), benefits of mammogram (6 items), barriers to mammogram (5 items), benefits of ultrasound (6 items), barriers to ultrasound (5 items), confidence (11 items), and health motivation (7 items). All items were formatted using an ordinal scale with a 5-point Likert scale response: 1 = "Strongly disagree," 2 = "Disagree," 3 = "Neutral," 4 = "Agree," and 5 = "Strongly agree" for positive statements. Each subscale can be utilized independently. However, when conducting an overall assessment of awareness regarding breast cancer and screening methods, the total score may be used. It's important to note that questions pertaining to barriers must be reversed before summing up the scores.

### Statistical analysis

The data were analysed using Stata version 15.0. Descriptive statistics, including mean, standard deviation (SD), frequency, and percentages, were used to describe the data. Internal consistency of the items within the health belief subscales was assessed using Cronbach's alpha. The association and comparison of items within the health belief subscales and across other variables were analysed using t-tests, analysis of variance (ANOVA), and linear regression models.

### Ethical approval and consent to participate

This study was conducted in accordance with the Declaration of Helsinki and under the review and approval of the Institutional Research Ethics Committee of the Faculty of Medicine, Chiang Mai University, Chiang Mai, Thailand (No. FAM 2564-08138) and Sanpatong Hospital Ethics Committee (No. SPT/REC 012/2564). All procedures were conducted following the relevant institutional guidelines and regulations.

## Results

### Distribution of sociodemographic factors of women (n = 130)

The sociodemographic characteristics of the 130 participants are presented in Table 1. The average age of the participants was 52.33 years (SD = 7.28). The majority of participants were single (61.54%). About 37.69% of the participants had attained a college-level education, while 51.54% had a monthly income exceeding 10,000 Baht (270 US dollars). Additionally, 41.54% of the participants had health insurance schemes through government or state enterprise officers.

**Table 1:** Sociodemographic variables of participants (N = 30).

Characteristic	Category	Frequency	%
Age	40–54	72	55.38
	55–70	58	44.62
Marital status	Married	50	38.46
	Single	80	61.54
Education	Primary or lower	42	32.31
	Secondary	39	30.00
	College	49	37.69
Monthly income	< 10,000 Baht*	63	48.46
	≥ 10,000 Baht	67	51.54
Health insurance schemes	Government or state enterprise officer	54	41.54
	Social security scheme	30	23.08
	Universal coverage scheme	46	35.38

*^1^Thai Baht (THB) equals 0.027 US dollars (USD).

### Distribution statistical data and Cronbach’s alphas for MT-CHBMS

Table 2 presents the mean ranged from 2.46 to 4.35 and SD ranged from 3.56 to 8.00. The overall Cronbach's alphas for the health belief model subscales were found to be within an acceptable range (0.70 or higher), indicating good internal consistency^40^.

**Table 2:** Means (M), Standard Deviations (SD), and Cronbach’s Alphas (α) for the Health Belief Model Subscales.

Scale	Min–Max	M (diving)	SD	α	Number of items
10. Motivation	21–35	30.45 (4.35)	3.56	0.85	7
4. Benefit-MG	14–30	25.36 (4.23)	3.90	0.94	6
7. Benefit-UTS	8–30	24.62 (4.10)	4.12	0.91	6
3. Benefit-BSE	8–30	24.32 (4.05)	3.86	0.88	6
5. Barrier-BSE	12–30	23.63 (3.94)	4.77	0.86	6
6. Barrier-MG	8–25	19.07 (3.81)	3.71	0.75	5
8. Barrier-UTS	10–25	19.04 (3.81)	3.77	0.80	5
9. Confidence	13–55	38.35 (3.49)	8.00	0.91	11
2. Seriousness	7–33	22.04 (3.15)	5.65	0.85	7
1. Susceptibility	5–23	12.29 (2.46)	4.89	0.93	5

*BSE* breast self-examination, *MG* mammogram, *UTS* ultrasound.

### Comparison of sociodemographic factors with MT-CHBMS

Table 3 presents the results of the statistical analyses conducted on various sociodemographic factors and their associations with the Health Belief Model subscales.

**Table 3:** Comparison of sociodemographic factors with health belief model subscales.

Parameter	Category	1. Susceptibility	2. Seriousness	3. Benefit-BSE	4. Benefit-MG	5. Barrier-BSE
Age	40–54	12.65 ± 4.71	22.03 ± 5.53	23.78 ± 4.06	25.03 ± 4.01	23.60 ± 4.40
	55–70	11.84 ± 5.11	22.05 ± 5.84	25.00 ± 3.52	25.78 ± 3.76	23.67 ± 5.22
	*t*	0.94	− 0.02	− 1.81	− 1.09	− 0.09
	*p*	0.351	0.981	0.073	0.279	0.929
Marital status	Married	12.62 ± 4.87	21.76 ± 5.47	24.42 ± 3.64	25.40 ± 3.68	23.70 ± 4.58
	Single	12.09 ± 4.92	22.21 ± 5.78	24.26 ± 4.02	25.34 ± 4.06	23.59 ± 4.91
	*t*	0.60	− 0.44	0.23	0.09	0.13
	*p*	0.548	0.658	0.822	0.93	0.897
Education	Primary or lower	12.69 ± 5.25	23.38 ± 5.66	24.93 ± 3.99	25.07 ± 4.46	22.88 ± 5.15
	Secondary	12.31 ± 4.33	22.59 ± 4.67	23.59 ± 3.95	25.31 ± 4.16	22.33 ± 4.65
	College	11.94 ± 5.06	20.45 ± 6.05	24.39 ± 3.67	25.65 ± 3.17	25.31 ± 4.07
	*F*	0.26	3.44	1.23	0.25	5.32
	*p*	0.768	0.035	0.296	0.776	0.006
Monthly income	< 10,000 Baht	12.29 ± 5.08	23.25 ± 5.37	24.35 ± 4.06	25.08 ± 4.13	22.49 ± 4.97
	≥ 10,000 Baht	12.30 ± 4.74	20.90 ± 5.70	24.30 ± 3.70	25.63 ± 3.68	24.70 ± 4.34
	*t*	− 0.02	2.43	0.08	− 0.80	− 2.71
	*p*	0.988	0.017	0.941	0.426	0.008
Health insurance schemes	Government or state enterprise officer	12.17 ± 5.10	21.35 ± 6.10	24.67 ± 3.77	26.19 ± 3.37	25.43 ± 4.34
	Social security scheme	11.23 ± 4.82	22.40 ± 4.87	23.60 ± 3.15	24.40 ± 4.28	21.37 ± 3.93
	Universal coverage scheme	13.13 ± 4.63	22.61 ± 5.59	24.39 ± 4.38	25.02 ± 4.11	22.96 ± 5.08
	*F*	1.41	0.69	0.74	2.34	8.50
	*p*	0.249	0.502	0.478	0.100	0.001

Parameter	Category	6. Barrier-MG	7. Benefit-UTS	8. Barrier-UTS	9. Confidence	10. Motivation
Age	40–54	18.86 ± 3.71	24.29 ± 3.97	19.17 ± 3.75	38.42 ± 7.90	30.18 ± 3.64
	55–70	19.33 ± 3.73	25.02 ± 4.29	18.88 ± 3.82	38.26 ± 8.18	30.78 ± 3.47
	*t*	− 0.71	− 0.99	0.43	0.11	− 0.95
	*p*	0.478	0.320	0.668	0.911	0.346
Marital status	Married	18.96 ± 3.64	24.56 ± 4.54	19.02 ± 3.51	38.02 ± 8.22	30.64 ± 3.67
	Single	19.14 ± 3.78	24.65 ± 3.86	19.05 ± 3.95	38.55 ± 7.91	30.33 ± 3.51
	*t*	− 0.26	− 0.12	− 0.04	− 0.37	0.49
	*p*	0.792	0.904	0.965	0.715	0.626
Education	Primary or lower	19.05 ± 3.57	24.60 ± 4.91	18.43 ± 3.79	39.10 ± 6.81	30.52 ± 3.16
	Secondary	18.69 ± 4.18	24.64 ± 3.95	18.72 ± 3.97	39.44 ± 7.45	30.00 ± 3.98
	College	19.39 ± 3.48	24.61 ± 3.54	19.82 ± 3.53	36.84 ± 9.20	30.73 ± 3.58
	*F*	0.38	0	1.75	1.43	0.47
	*p*	0.686	0.999	0.178	0.244	0.625
Monthly income	< 10,000 Baht	18.59 ± 3.63	24.35 ± 4.50	18.16 ± 3.81	39.16 ± 7.20	30.37 ± 3.40
	≥ 10,000 Baht	19.52 ± 3.76	24.87 ± 3.73	19.87 ± 3.57	37.58 ± 8.67	30.52 ± 3.74
	*t*	− 1.44	− 0.71	− 2.64	1.12	− 0.25
	*p*	0.152	0.477	0.009	0.263	0.803
Health insurance schemes	Government or state enterprise officer	19.83 ± 3.44	25.06 ± 3.84	20.28 ± 3.53	37.52 ± 9.20	30.72 ± 3.63
	Social security scheme	17.10 ± 3.98	24.57 ± 3.78	17.23 ± 3.58	38.63 ± 6.45	30.27 ± 3.85
	Universal coverage scheme	19.41 ± 3.51	24.13 ± 4.64	18.76 ± 3.71	39.13 ± 7.45	30.24 ± 3.35
	*F*	5.94	0.63	6.85	0.53	0.27
	*p*	0.003	0.536	0.002	0.593	0.760

Participants with education less than secondary school exhibited higher scores in the Seriousness subscale compared to other education level groups (*F* = 3.44, *p* = 0.035). Participants with a college educational level had higher scores in the Barrier-BSE subscale compared to other education level groups (*F* = 5.32, *p* = 0.006).

In terms of monthly income, participants in the lower 10,000 Baht income group demonstrated higher scores in the Seriousness subscale compared to the more than 10,000 Baht income group (*t* = 2.43, *p* = 0.017). Conversely, the more than 10,000 Baht income group had higher scores in the Barrier-BSE and Barrier-UTS subscales compared to the lower 10,000 Baht income group (*t* =  − 2.71, *p* = 0.008 and *t* =  − 2.64, *p* = 0.009).

Participants with health insurance schemes through government or state enterprise officer schemes exhibited higher scores in the Barrier-BSE and Barrier-UTS subscales compared to other groups (*F* = 8.50, *p* = 0.001 and *F* = 6.85, *p* = 0.002). Additionally, participants with health insurance schemes through government or state enterprise officer schemes and those covered under the universal coverage scheme had higher scores in the Barrier-MG subscale compared to the social security scheme group (*F* = 5.94, *p* = 0.003).

### Multiple linear regression model of MT-CHBMS

Table 4 presents the results of multiple linear regression analysis. None of the factors were found to be significant associated with of Seriousness subscale. However, health insurance schemes were found to be a significant associated with of the Benefit-MG and Barrier-BSE (β_m_ =  − 2.48, P = 0.023 and β_m_ =  − 3.38, P = 0.008, respectively). Both monthly income and health insurance schemes were significant associated with of the Barrier-MG and Barrier-UTS (β_m_ = 2.65, P = 0.008, β_h_ =  − 3.11, P = 0.002 and β_m_ = 2.49, P = 0.013, β_h_ =  − 3.40, P = 0.001, respectively).

BM= item from benefit to mammogram, BARB = item from barrier to breast self-examination, BARM = item from barrier to mammogram, BAU = item from barrier of ultrasound.

**Table 4:** Multiple linear regression model of health belief model subscales.

Parameter	Category	2. Seriousness			4. Benefit-MG			5. Barrier-BSE			6. Barrier-MG			8. Barrier-UTS		
		B	SE	p-value	B	SE	p-value	B	SE	p-value	B	SE	p-value	B	SE	p-value
Education	Primary or lower	Ref			Ref			Ref			Ref			Ref		
	Secondary	− 0.25	1.37	0.855	0.43	0.96	0.655	− 0.54	1.11	0.628	− 0.57	0.87	0.517	− 0.03	0.87	0.970
	College	− 2.58	1.87	0.168	− 0.96	1.30	0.464	− 0.04	1.51	0.976	− 1.97	1.18	0.098	− 1.50	1.18	0.207
Monthly	< 10,000	Ref			Ref			Ref			Ref			Ref		
income	> = 10,000	− 1.84	1.56	0.240	0.41	1.09	0.708	2.10	1.26	0.099	2.65	0.99	0.008	2.49	0.99	0.013
Health insurance schemes	Government or state enterprise officer	Ref			Ref			Ref			Ref			Ref		
	Social security scheme	− 0.65	1.54	0.673	− 2.48	1.08	0.023	− 3.38	1.25	0.008	− 3.11	0.97	0.002	− 3.40	0.98	0.001
	Universal coverage scheme	− 1.95	1.65	0.234	− 1.73	1.15	0.133	− 0.74	1.33	0.579	0.20	1.04	0.851	− 0.85	1.04	0.416
	Constant	24.88	1.51		26.56	1.06		23.72	1.22		19.25	0.96		19.41	0.96	

*B* unstandardized coefficient, *SE* standard error, *MG* mammogram, *BSE* breast self-examination, *UTS* ultrasound.

### Comparison of monthly income and health insurance schemes with the significant subscales of MT-CHBMS

To delve deeper into the specifics, each subscale item, including those related to the benefits and barriers of mammograms, breast self-examination, and ultrasound, was compared among different monthly income groups and health insurance schemes using t-tests and ANOVA analyses (Table 5). For the Barrier-BSE subscale, the group with an income of 10,000 Baht or more demonstrated higher scores in Barrier-BSE compared to the less than 10,000 Baht income group across the BARB1 (funny), BARB3 (embarrassing), and BARB5 (unpleasant) items. Additionally, participants with health insurance schemes through government or state enterprise officer schemes exhibited higher scores in Barrier-BSE compared to other groups across all BARB (1–6) items.

**Table 5:** Comparison of Monthly Income and Health insurance schemes with the significant Subscales of MT-CHBMS.

	Monthly income (Baht)		*p*-value	Health insurance schemes			*p*-value
	< 10,000	≥ 10,000		Government or state enterprise officer	Social security scheme	Universal coverage scheme	
4. Benefit-MG							
BM1	4.16 ± 0.65	4.34 ± 0.59	0.093	4.39 ± 0.56	4.13 ± 0.68	4.17 ± 0.64	0.112
BM2	4.21 ± 0.74	4.30 ± 0.65	0.453	4.41 ± 0.60	4.13 ± 0.78	4.15 ± 0.73	0.105
BM3	4.21 ± 0.74	4.37 ± 0.71	0.195	4.44 ± 0.69	4.10 ± 0.71	4.24 ± 0.77	0.097
BM4	4.24 ± 0.82	4.22 ± 0.78	0.919	4.33 ± 0.75	4.03 ± 0.81	4.24 ± 0.82	0.252
BM5	4.11 ± 0.84	4.13 ± 0.81	0.874	4.26 ± 0.76	3.93 ± 0.87	4.09 ± 0.86	0.209
BM6	4.16 ± 0.77	4.25 ± 0.79	0.486	4.35 ± 0.76	4.07 ± 0.78	4.13 ± 0.78	0.191
Sum	25.08 ± 4.13	25.63 ± 3.68	0.426	26.19 ± 3.37	24.40 ± 4.27	25.02 ± 4.11	0.100
5. Barrier-BSE							
BARB1	3.94 ± 1.13	4.36 ± 0.79	0.015	4.48 ± 0.75	3.87 ± 0.94	3.96 ± 1.17	0.006
BARB2	3.33 ± 1.20	3.64 ± 0.93	0.104	3.81 ± 1.03	3.20 ± 0.81	3.30 ± 1.21	0.014
BARB3	3.98 ± 1.07	4.40 ± 0.78	0.012	4.46 ± 0.88	3.93 ± 0.87	4.07 ± 1.02	0.024
BARB4	3.87 ± 1.08	4.10 ± 1.03	0.215	4.26 ± 0.94	3.47 ± 1.11	4.02 ± 1.06	0.004
BARB5	3.52 ± 1.12	4.03 ± 0.95	0.006	4.06 ± 1.04	3.40 ± 0.93	3.72 ± 1.11	0.021
BARB6	3.84 ± 1.05	4.16 ± 1.02	0.078	4.35 ± 0.93	3.50 ± 0.97	3.93 ± 1.08	0.001
Sum	22.49 ± 4.97	24.7 ± 4.34	0.008	25.43 ± 4.34	21.37 ± 3.93	22.96 ± 5.08	0.001
6. Barrier-MG							
BARM1	3.60 ± 1.13	3.90 ± 1.00	0.121	4.00 ± 0.97	3.33 ± 1.12	3.74 ± 1.08	0.023
BARM2	4.11 ± 0.95	4.37 ± 0.81	0.093	4.52 ± 0.67	3.77 ± 1.17	4.24 ± 0.79	0.001
BARM3	3.84 ± 1.08	4.03 ± 0.92	0.285	4.13 ± 0.89	3.57 ± 1.10	3.96 ± 1.01	0.046
BARM4	3.71 ± 1.04	3.66 ± 1.14	0.764	3.61 ± 1.20	3.40 ± 1.00	3.96 ± 0.94	0.074
BARM5	3.32 ± 1.25	3.57 ± 1.13	0.235	3.57 ± 1.18	3.03 ± 1.19	3.57 ± 1.19	0.097
Sum	18.59 ± 3.63	19.52 ± 3.76	0.141	19.83 ± 3.44	17.1 ± 3.98	19.41 ± 3.51	0.003
8. Barrier-UTS							
BAU1	3.35 ± 1.23	3.81 ± 0.93	0.018	3.80 ± 1.05	3.37 ± 0.96	3.48 ± 1.22	0.168
BAU2	3.92 ± 1.04	4.27 ± 0.73	0.028	4.43 ± 0.69	3.50 ± 1.01	4.11 ± 0.88	0.001
BAU3	3.83 ± 1.01	4.13 ± 0.81	0.056	4.22 ± 0.84	3.70 ± 0.92	3.89 ± 0.97	0.031
BAU4	3.87 ± 1.02	3.99 ± 0.88	0.504	4.11 ± 0.88	3.53 ± 0.97	3.98 ± 0.95	0.025
BAU5	3.19 ± 1.20	3.67 ± 1.11	0.019	3.72 ± 1.17	3.13 ± 1.11	3.30 ± 1.17	0.054
Sum	18.16 ± 3.81	19.87 ± 3.57	0.009	20.28 ± 3.53	17.23 ± 3.58	18.76 ± 3.75	0.002

*BM* item from benefit to mammogram, *BARB* item from barrier to breast self-examination, *BARM* item from barrier to mammogram, *BAU* item from barrier of ultrasound.

Regarding the Barrier-MG subscale, participants with health insurance schemes through government or state enterprise officer schemes had higher scores in Barrier-MG compared to other groups across the BARM1 (worry), BARM2 (embarrassing), and BARM3 (take too much time) items.

In terms of the Barrier-UTS subscale, the group with an income of 10,000 Baht or more demonstrated higher scores in barrier-UTS compared to the less than 10,000 Baht income group across the BAU1 (worry), BAU2 (embarrassing), and BAU5 (cost too much money) items. Additionally, participants with health insurance schemes through government or state enterprise officer schemes had higher scores in Barrier-UTS compared to other groups across the BAU2 (embarrassing), BAU3 (take too much time), and BAU4 (painful) items.

## Discussion

The objective of the study was to investigate differences in beliefs related to breast examination among various sociodemographic variables in Thai women, and the results have confirmed their presence.

Using multiple linear regression analysis with the MT-CHBMS, the results indicated several findings. Health insurance schemes were associated with Benefit-MG, Barrier-BSE, Barrier-MG and Barrier-UTS subscales. Additionally, monthly income showed associations with the Barrier-MG and Barrier-UTS subscales. The most common barriers reported by participants were feeling “embarrassed”, “worry”, and feeling that it “takes too much time”.

Unlike population-based studies, the current study reveals a distinct finding: health beliefs were not associated with age, marital status, and education. This contrasts with findings from other related studies, such as those involving Turkish and Iranian women, where age, marital status, and education were significantly correlated with health beliefs scales.^41,42^.

Interestingly, our study observed that distinct income groups were associated with varying outcomes in the Barriers-MG and Barriers-UTS subscales. Notably, there is a dearth of similar literature available for direct comparison. However, Kirag and Kizilkaya et al.^35^ reported correlations between income levels and Benefit-BSE, Barriers-BSE, Self-efficacy, and Benefit MG, while Altunkurek and Hassan Mohamed^37^ also identified a relationship between income status and the Susceptibility and Health Motivation subscales. The connection between lower income and barriers to BSE is not easily explained. It is possible that there are intermediary variables requiring further investigation.

According to the Health Belief Model, perceived barriers have consistently been identified as the most influential predictor in various studies for practicing BSE and mammography^43^. Recent studies have also shown that perceiving more benefits, having higher confidence, and experiencing fewer barriers are positively associated with BSE practice^16,44,45^. Similarly, perceiving more benefits and fewer barriers is positively associated with mammography^44^. In this study, it was found that the social security scheme associated with Barrier-BSE, Barrier-MG and Barrier-UTS. In addition, the social security scheme had lower scores than the government or state enterprise officer and universal coverage scheme in the barrier to BSE, barrier to mammogram, and barrier to ultrasound subscales. It is to note that the government or state enterprise officer scheme beneficiaries benefit from a higher level of healthcare coverage compared to the other two schemes. It offers a high level of coverage and includes access to government hospitals and medical facilities. This scheme beneficiaries typically have access to a comprehensive range of medical services, often with little or no out-of-pocket expenses. The scheme provides coverage for both routine healthcare and specialized treatments, including access to government-run healthcare facilities. The social security scheme members often enjoy relatively comprehensive healthcare benefits, and the quality of care is generally good. However, it is limited to formal sector employees and their dependents, which means that informal sector workers and those not covered by formal employment arrangements are not eligible. The universal coverage scheme aims to provide equitable access to healthcare for all, emphasizing the principle of social justice. The scheme may have limitations on specialized or high-cost medical treatments, and there may be variations in the quality of care among different facilities.

The impact of the healthcare scheme type on barriers to BSE, MG, or UTS may be influenced by numerous factors. Nevertheless, the results suggests that women who have health coverage through the social security scheme may benefit from targeted interventions to improve detection. Evidence for program planning should be implement in health insurance schemes groups such as health education, skill training and confidence in performing for BSE, reminders to perform BSE, regular use of BSE record booklets^15,46^.

One of the general barriers observed in this study is the lack of knowledge and awareness of breast cancer among the participants, as evidenced by their low scores in the Susceptibility, Seriousness, and Confidence scales. Knowledge is identified as the most influential barrier affecting the engagement of participants in BSE, particularly in low to middle-income countries and rural areas where resources are limited^47^. Participants in this study perceived their ability to perform the BSE technique as low, indicating a lack of knowledge or a lack of regular practice. Susceptibility refers to participants' perception of their chances of being at risk for a disease. In this study, participants perceived their chances of having a risk or disease as low, indicating a potential lack of knowledge regarding the risk factors of breast cancer, such as young age, no family history of cancer, and the absence of breast lumps. Seriousness pertains to participants' perception of the severity of the consequences associated with the disease. In this study, participants may perceive breast cancer as not causing pain, exhibiting no symptoms or signs, and not posing a significant threat. This suggests a lack of knowledge or the use of defence mechanisms such as denial or rationalization, similar to behaviours observed in smokers and alcohol drinkers^48,49^. Consistent with many Thai studies, interventions focusing on health education and skill training for BSE are recommended to address these knowledge gaps^17,21,46^.

One of the most common barriers to early screening detection identified in this study is the feeling of “embarrassment” and “worry”. Similar to Amin MN et al.^50^, this study conducted a hospital survey. The feeling of embarrassment can be considered a cultural barrier, where women may feel too embarrassed to have their breasts examined by a male doctor. This cultural aspect can hinder their willingness to seek medical attention for abnormalities. Worry, on the other hand, is associated with feelings of anxiety. Women may experience worry related to breast lumps, the potential consequences of breast cancer, and concerns about health professionals and healthcare facilities. Additionally, the perception that screening “takes too much time” can be a deterrent. Women may feel that they are too busy, have limited time, or believe that they lack sufficient time to perform BSE and undergo screening procedures^47^. Interventions should focus on problem-solving approaches and aim to improve healthcare services in order to overcome barriers faced by the participants. By addressing these barriers and concerns, healthcare providers can create a more supportive and comfortable environment for women to engage in early screening and detection practices. Apart from the issue of “embarrassment”, “worry”, and “takes too much time”, which should be considered as one of the barriers to BSE, mammograms, and ultrasounds, there could be other contributing factors. Future research should incorporate qualitative studies to explore additional causal factors influencing the practice or non-practice of BSE, as well as the utilization or non-utilization of mammograms and ultrasounds. Additionally, it is recommended to compare interventions using a before-and-after study design involving the three main public health insurance schemes: government or state enterprise officer, social security scheme, and universal coverage scheme. This examination is necessary to identify effective interventions for women within each health insurance scheme who may face different barriers.

Participants in this study are to be more empowering their health. They have the highest score of Health Motivation and comparing Benefit-MG and Benefit-UTS more than Benefit-BSE. Conversely, Barrier-BSE when comparing Barrier-MG and Barrier-UTS is inverse. This is show that they would like to take investigate accuracy screening tools more than their manual. As health practitioners’ perspective of Thai study would like to drive a policy of national cancer act to enable women’s rights for accessing standardized screening tools^10^.

### Evidence for planning and future research

There is associated between a monthly income and perceived Barriers-MG and Barriers-UTS. This predictor may be sensitive and difficult to approach regarding their monthly income when implementing intervention strategies targeting MG and UTS promotion. However, there is health insurance schemes which associated with Benefit-MG, Barrier-BSE, Barrier-MG and Barrier-UTS subscale. Also, health insurance schemes in the social security scheme is the predictor of perceived Barrier-BSE, Barrier-MG and Barrier-UTS. Specifically, the perceived barriers subscale can help identify the problems of implementation. Furthermore, attitudes toward BSE, mammograms, and ultrasounds can be compared in terms of their benefits and barriers. Such comparisons can yield valuable insights for the development of targeted interventions and approaches aimed at increasing breast cancer screening among Northern Thai women in a hospital-based setting. The design of programs and future research should take this evidence into account during implementation. Future research could employ a before-and-after study design, integrating health education and skill training for BSE, and incorporating qualitative studies to explore the additional causal factors influencing the practice or non-practice of BSE, using or non-using mammogram/ultrasound. Moreover, investigating how to improve healthcare services to ensure women's satisfaction would be beneficial.

### Strength and limitations

This study is the first research project known to utilize the MT-CHBMS to study the association between sociodemographic factors and health beliefs of breast cancer and screening behaviors. Additionally, the inclusion of new items related to ultrasound in the MT-CHBMS holds promise for the assessment of breast cancer beliefs among Thai women with dense breast masses and the potential integration of advanced technologies such as artificial intelligence in the future.

However, it is important to acknowledge the limitations of this study. Firstly, the cross-sectional design employed cannot establish causal relationships between beliefs and screening practices. Secondly, the results may not be generalizable to the entire population due to the selection of participants from a single geographic area and hospital setting in Northern Thailand. Thirdly, convenience sampling may cause these study results to only generalize to this research's sampling group. Fourthly, small sample size may cause low statistical power, increased error rate, and less precise information. Fifthly, structured interviews may be subject to interviewer or social desirability bias. Sixthly, no external validation, e.g., concurrent validity, was conducted along with the construct validity. Test–retest reliability and predictive validity were not examined and should be included in future research. Lastly, certain factors such as family history of breast cancer and other breast masses were not specifically excluded from the study, which could potentially influence participants' beliefs regarding breast cancer and their practices related to screening methods.

## Conclusion

This study marked the first use of the MT-CHBMS to investigate the association between sociodemographic factors and health beliefs related to breast cancer screening. The findings provide evidence for program design and future research aimed at increasing breast cancer screening among women in Northern Thailand in a hospital-based setting. By successfully implementing the interventions, the ssocial security scheme represents the most targeted interventions can serve as role models for other health insurance schemes and contribute to enhancing the effectiveness of screening among women.

## Data Availability

The datasets used and/or analyzed during the current study are available from the corresponding author on reasonable request.
